# Phocid Seal Leptin: Tertiary Structure and Hydrophobic Receptor Binding Site Preservation during Distinct Leptin Gene Evolution

**DOI:** 10.1371/journal.pone.0035395

**Published:** 2012-04-19

**Authors:** John A. Hammond, Chris Hauton, Kimberley A. Bennett, Ailsa J. Hall

**Affiliations:** 1 Institute for Animal Health, Compton, Newbury, UK; 2 Ocean and Earth Science, University of Southampton, National Oceanography Centre – Southampton, Southampton, United Kingdom; 3 Marine Biology and Ecology Research Centre, School of Marine Science and Engineering, Plymouth, Plymouth University, United Kingdom; 4 Sea Mammal Research Unit, Scottish Oceans Institute, University of St Andrews, St Andrews, United Kingdom; California State University Fullerton, United States of America

## Abstract

The cytokine hormone leptin is a key signalling molecule in many pathways that control physiological functions. Although leptin demonstrates structural conservation in mammals, there is evidence of positive selection in primates, lagomorphs and chiropterans. We previously reported that the leptin genes of the grey and harbour seals (phocids) have significantly diverged from other mammals. Therefore we further investigated the diversification of leptin in phocids, other marine mammals and terrestrial taxa by sequencing the leptin genes of representative species. Phylogenetic reconstruction revealed that leptin diversification was pronounced within the phocid seals with a high dN/dS ratio of 2.8, indicating positive selection. We found significant evidence of positive selection along the branch leading to the phocids, within the phocid clade, but not over the dataset as a whole. Structural predictions indicate that the individual residues under selection are away from the leptin receptor (LEPR) binding site. Predictions of the surface electrostatic potential indicate that phocid seal leptin is notably different to other mammalian leptins, including the otariids. Cloning the grey seal leptin binding domain of LEPR confirmed that this was structurally conserved. These data, viewed *in toto*, support a hypothesis that phocid leptin divergence is unlikely to have arisen by random mutation. Based upon these phylogenetic and structural assessments, and considering the comparative physiology and varying life histories among species, we postulate that the unique phocid diving behaviour has produced this selection pressure. The Phocidae includes some of the deepest diving species, yet have the least modified lung structure to cope with pressure and volume changes experienced at depth. Therefore, greater surfactant production is required to facilitate rapid lung re-inflation upon surfacing, while maintaining patent airways. We suggest that this additional surfactant requirement is met by the leptin pulmonary surfactant production pathway which normally appears only to function in the mammalian foetus.

## Introduction

Leptin is a multifunctional protein involved in numerous physiological processes including energy regulation, haematopoiesis, inflammation, foetal development, puberty and digestion [1], [2], [3]. Produced mainly by adipocytes leptin is primarily viewed as being involved in energy balance and as a mediator of the adaptation to fasting [1]. Increased levels in the blood positively correlate with fat stores in many species [4], [5], [6]. In terrestrial mammals, leptin provides a local and central feedback signal to regulate the size of energy stores. It stimulates the mobilization of fat from surrounding adipocytes for use as a metabolic fuel and acts on appetite centres in the brain to reduce food intake and alter preference for fats. It is now clear that leptin is also fundamental to the structural and functional development and homeostasis of the mammalian lung [7], [8], where it appears to be an important growth factor [9], [10] and signalling hormone in the pulmonary surfactant production pathway [7]. The evolution of marine mammals and their consequent life history and physiological traits suggests, from our knowledge of the functions of leptin, that this signalling protein is likely to play a key role. For example, all marine mammals store energy as a subcutaneous layer of fat (blubber), utilizing it during periods of prolonged fasting (such as migration, breeding and moulting). They therefore need to be able to regulate their fat stores, energy acquisition needs and periods of fasting very well, a requirement in which leptin may conceivably play a role. In addition they are able to dive repeatedly, to sometimes considerable depths, equipped with essentially the same respiratory mechanisms as terrestrial mammals which means they have evolved mechanisms to cope with high and consistently variable changes in respiratory pressure and volume.

Within the marine mammal suborder Pinnipedia, the earless or true seals (Family Phocidae) possess unique and exclusive physiological traits. They are capital breeders that mobilize stored fat annually for milk production during lactation. They are well adapted to tolerate this and other prolonged periods of fasting, during which they *usually* rely on their blubber for energy. In contrast, the sea lions and fur seals (Family Otariidae) must feed during lactation and are known as income breeders. Phocid seals are also the only group of marine mammals to regularly dive on expiration, allowing their lungs to collapse on each dive and then reinflating them on inspiration at surfacing. They have the least modified airways of all the marine mammals [11], [12] yet include the deepest diving seal species, such as the Southern elephant (*Mirounga leonina*) and Weddell seals (*Leptonychotes weddellii*), which can withstand the hydrostatic pressure at depths of between ∼600 and ∼2000 m respectively [13], [14]. In contrast, all other marine mammal Families have highly modified airways compared to their terrestrial counterparts [11]. Otariids and cetaceans are known to dive on inspiration, which helps to minimize the potential for nitrogen narcosis [15]. Thus diving behaviour has also created a strong selective pressure on lung function and physiology which has, however, led to the evolution of patently different solutions in the different marine mammal Families.

The phocid leptin gene is expressed in the blubber, bone marrow and lung of phocids. Together with evidence of protein translation and in combination with the presence of parathyroid hormone related peptide (PTHrP), another surfactant pathway protein [7], we have previously argued that leptin may play an additional role in the lung physiology of adult phocid seals. By providing the means for the additional surfactant production required for lung compliance during repeated re-inflation following dives [16] leptin synthesis in phocid lung cells is maintained and not down-regulated at birth [7].

Despite the structural constraints of receptor interaction, there is evidence of both purifying and positive selection of leptin genes in different mammalian groups [17], [18], [19]. Recently it was demonstrated that adaptive evolution has occurred in pika (order Lagomorpha) leptin as an ecological adaptation to extreme environmental stress [19]. Evidence of positive selection of leptin has also been reported in heterothermic bats (order Chiroptera) that undergo periods of daily torpor or hibernation [18]. We have previously shown that the leptin genes in the grey (*Halichoerus grypus*) and harbour seal (*Phoca vitulina*) have significantly diverged from those of other mammals and recent evidence has indicated that this divergence has been driven by positive selection [16], [20]. It is therefore possible that the unique environment these animals have evolved to inhabit has created diversifying selection that has driven the adaption of phocid leptin.

Cetaceans and pinnipeds returned to the aquatic environment as separate lineages. Although the pinnipeds have retained some terrestrial behaviour, spending the majority of their lives at sea and some periods on land, cetaceans have developed an entirely aquatic lifestyle. The phylogenetic relationship among the marine mammals has been extensively studied using complete mitochondrial genome sequences. The cetaceans are nested within the Artiodactyla, a group not now considered to be monophyletic, with the cetaceans being more closely related to the hippopotamus than the cows, pigs and camels [21]. The Certartiodactyla (whales, dolphins and even-toed ungulates) were estimated to have diverged from the Carnivora, which includes the pinnipeds, canids and felids, around 76 mya. The divergence between ruminant artiodactyls and cetaceans is estimated to be about 60 mya and the divergence between pinnipeds and caniforms about 50 mya. The basal split between the phocids and otariids occurred about 33 mya [22], [23]. These independent lineages have subsequently evolved different respiratory adaptations, diving capabilities and life history strategies. However, it has been uncertain whether the divergence that we reported previously for grey and harbour seals is consistent for all Families of marine mammals or whether it represents evidence for positive selection exclusively within the true seals.

Herein we report the results of a phylogenetic analysis of leptin evolution within mammalian lineages to investigate the hypothesis that positive selection has occurred within marine mammals and the likely consequences on leptin function. Leptin gene phylogeny has been combined with predictions of tertiary structure within the leptin molecule and a study of sequence conservation within the leptin receptor (LEPR) gene to assess the evidence for the conservation of structure and function within different marine and terrestrial mammal groups. We conclude that there is significant evidence to support a hypothesis of positive selection of the leptin gene within the phocid seals, but not within cetaceans. Selection in phocids has taken place at sites away from the LEPR binding site. Structural predictions, based on the crystal structure of human leptin, support the conservation of a hydrophobic LEPR binding cleft in all modelled leptin molecules but with differences in the surface electrostatic potential that are apparently unique to the Phocidae. The exclusive physiological adaptations, particularly to diving on expiration, are considered to have produced this positive selection of the leptin gene, unique to the true seals.

## Results

### Isolation of pinniped and cetacean leptin sequences

**Figure 1:**
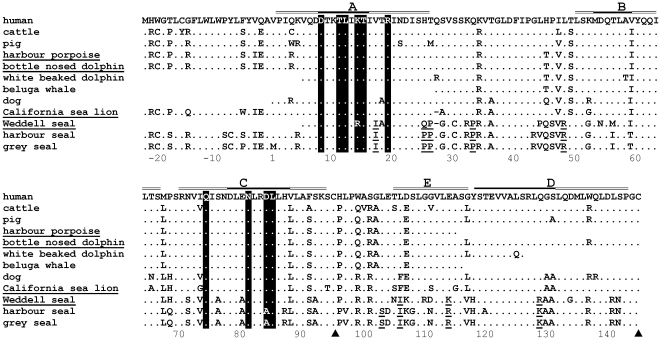
Leptin LEPR-binding residues are conserved across diverse mammalian phyla including the marine mammals. Summary representative alignment of the predicted amino acid sequences of selected mammalian leptin molecules. Identical residues to human leptin are marked with a dot, spaces manually introduced into the alignment are indicated by ‘-’. Shading represents the leptin residues critical for LEPR binding [24], [25], underlined residues show evidence of positive selection within representatives of the Family Phocidae. The cysteine residues critical for folding are indicated by ▴. The residues that form the helices in the human leptin are marked with a track above the alignment, with the most conserved regions filled. Species for which leptin sequences are reported in this study are underlined. Residue numbering is based on the mature protein from human, residues with a negative placement occur within the signal sequence of the nascent peptide.

Leptin has been structurally conserved throughout mammalian evolution as it is an essential and multifunctional protein. Our previous work [16] showed that leptin from phocid seals within the Phocinae subfamily possessed a number of non-synonymous substitutions in regions that are generally conserved in other mammalian species, including within the four predicted α-helical structures [16]. To determine how restricted this divergence is among the pinnipeds, we cloned the leptin gene product from the other subfamily of phocids, the Monachinae, represented by the Weddell seal. The California sea lion (*Zalophus californianus*) leptin cDNA was also cloned to represent a non-phocid species within the pinniped family. A complete leptin sequence was cloned from the California sea lion and a partial sequence was obtained from the Weddell seal that includes all but the first five residues of the mature protein (Fig. 1). Both these pinniped leptin molecules were predicted to encode a functional leptin protein and contained the cysteine residues that are essential to maintain tertiary structure [24] (Fig. 1).

Two cetacean leptin cDNA sequences were also obtained; cetaceans are a distantly related group of marine mammals to the pinnipeds with different physiological and behavioural adaptations. A complete harbour porpoise (*Phocoena phocoena*) sequence was cloned and the bottlenose dolphin (*Tursiops truncatus*) sequence was predicted from the genome Contig465483 (as of April 19^th^ 2007) available from the Baylor College of Medicine Human Genome Sequencing Centre (http://www.hgsc.bcm.tmc.edu/project-species-m-Dolphin.hgsc?pageLocation=Dolphin). Each of these leptin molecules was also predicted to code for a functional leptin and contained the cysteine residues essential for tertiary structure formation (Fig. 1).

### Phylogenetic analysis of marine mammal leptins and evidence for positive selection

The leptin sequences obtained in this study were aligned with publically available mammalian leptin cDNA sequences (Figure S1). All available species within the same order and suborders as the pinnipeds and the cetaceans were included. An illustrative version of this alignment is shown in Fig. 1, with species selected to represent both related and unrelated species to those cloned in this study. The vast majority of the unique substitutions that were previously found in the grey and harbour seal are also present in the Weddell seal. In contrast, the California sea lion shows a much higher identity to other carnivore relatives such as the dog (90%), and indeed to other unrelated mammals such as cattle (86.5%) than to the phocid seal species with which it shares a more recent ancestor (69.5–70.2%).

**Figure 2:**
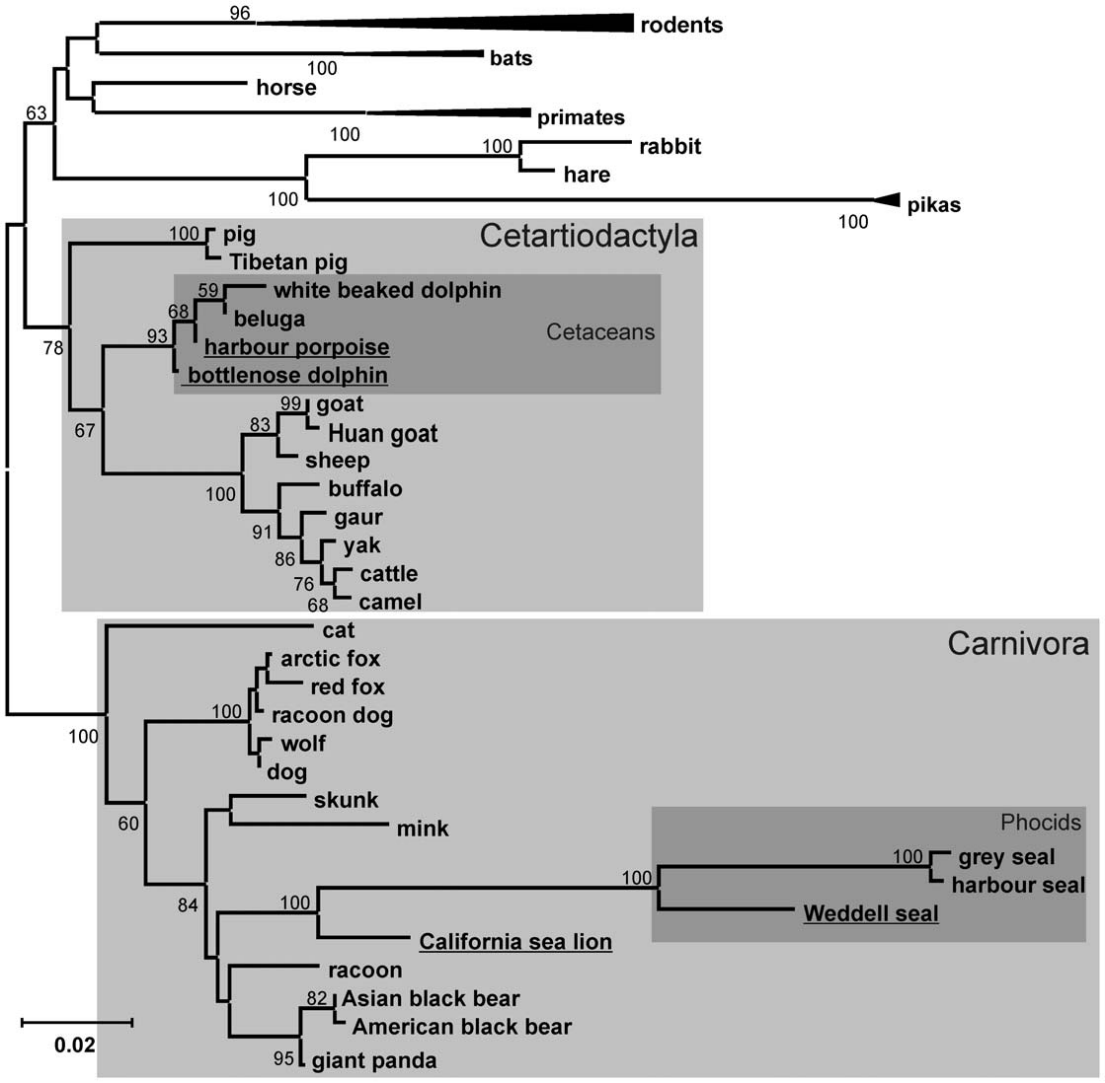
Pinniped and cetacean leptin sequences clade within the Carnivora and Cetartiodactyla respectively. Neighbour-joining (NJ) analysis, using the Tamura 3-parameter method with 1000 replicates, comparing diverse nucleotide sequences of mammalian leptin sequences with the sequences isolated in this study (underlined). The tree was rooted at the midpoint and support for each node (expressed as a percentage) is shown when >50%. The rodent, primate and lagomorphs clades have been collapsed for clarity. Evidence of positive selection was detected along the branch leading to the phocids indicated with ‘*’. Consistent topologies were predicted using Neighbour-joining and Maximum Likelihood methods (data not shown – see Methods for description).

The entire alignment was used to generate phylogenetic predictions which have been summarised as a representative NJ tree shown in Fig. 2. Maximum likelihood and Neighbour-Joining methods were used with several different models but no significant differences in tree topology were observed. Only minor and poorly supported relationship changes between primates and rodents were seen. Figure 2 supports the position of both the seal and cetacean sequences within their appropriate clades. The comparatively long branch length leading to the phocid seals demonstrates the large number (43) of substitutions between these and the otariid species, including the California sea lion, as well as the other carnivore species. Otariids and phocids shared a common ancestor approximately 33 mya, and both diverged from a common terrestrial ancestor approximately 45 mya [21]. The cetaceans shared a common ancestor with the other members of the Cetartiodactyla approximately 60 mya [23]. These topology predictions all support the rapid rate of leptin divergence since these groups of pinnipeds diverged. Similar high rates of leptin sequence divergence have recently been reported in beavers, pikas, marmosets and heterothermic bats. It is also clear from Fig. 2 that sequences from pikas and bats also have a large number of substitutions compared to their closest relative [17], [18].

**Figure 3:**
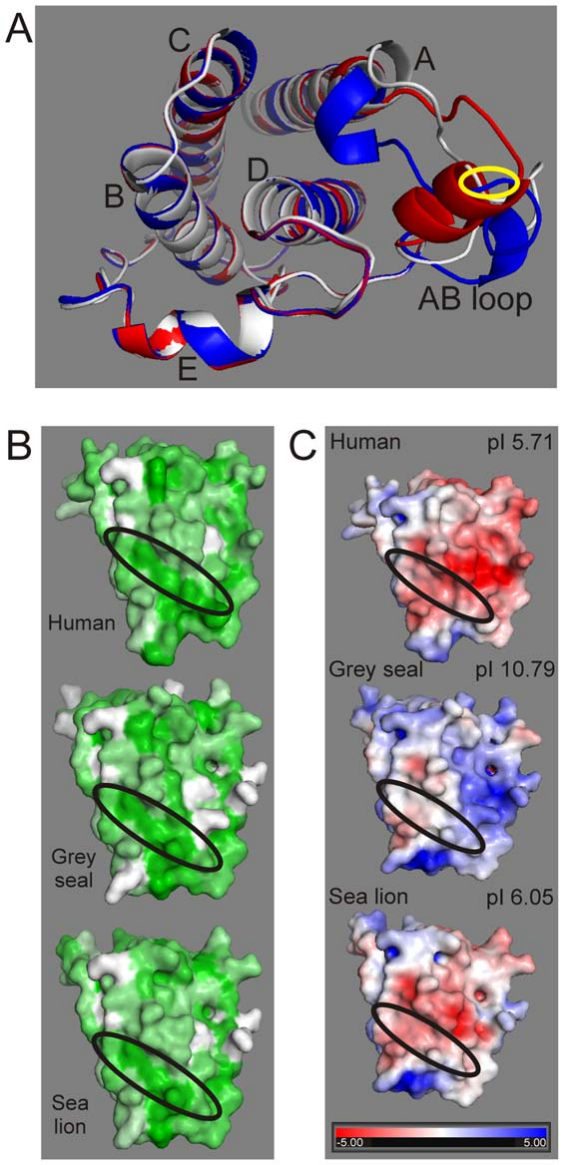
Conserved tertiary structure within mammalian leptins. (A) Ribbon models based on complete human (white, PDB coordinate file kindly provided by Prof. F. Zhang), grey seal (blue), and California sea lion (red) models are overlaid and show the conserved tertiary structure of the Helices A to E and the position of the variable AB loop. Structural prediction based on the crystallographic structure for human leptin, PDB ID: 1AX8, using SWISS-Model [38]. The position of the additional cysteine (Cys^31^) predicted in the phocid seal sequence is identified by the yellow ellipse within the AB loop; this substitution has no significant effect on the overall tertiary structure of the molecule. (B) Conservation of the hydrophobic leptin receptor (LEPR)-binding cleft across terrestrial and marine mammal leptin molecules. Figure shows the predicted surface hydrophobicity for human, grey seal and California sea lion (green = hydrophobic, white = hydrophilic) with the binding cleft circled by a black ellipse in each image. (C) Distribution of surface electrostatic potential of phocid seal leptin is markedly different to that of other marine and terrestrial mammals, especially away from the hydrophobic LEPR binding cleft. Figure shows Poisson–Boltzmann electrostatic surface potential of solvent-exposed surfaces coloured from red (−5.0) to blue (+5.0) for predicted leptin structures for the human, grey seal and California sea lion as well as the predicted isoelectric point (pI) for each molecule. Residue substitution in grey seal leptin produces a more neutral hydrophobic cleft and a considerably more electropositive surface elsewhere on the molecule than in the case of the human and California sea lion.

As reported, class 1 helical cytokines including leptins do show divergence in their primary structure in vertebrates, whilst remaining conserved in their overall predicted secondary and tertiary structure [17]. The predicted helices are crucial to the tertiary structure of the leptin molecule and, as the two cysteines at positions 96 and 146 that form a disulphide bond key to the structure of human leptin are both intact [24], the phocid and otariid leptins are predicted to be structurally conserved (Fig. 3A). Although an additional cysteine is predicted at position 31 within the phocid seal leptins, it occurs within the AB loops of each structure, and therefore has very little influence on the predicted tertiary structure (Fig. 3A). Several substitutions are identified at the predicted junction between the two coding exons at the end of helix A (Fig. 1), but alignment with the dog genomic sequence confirms that no intron sequence forms part of the mature phocid cDNA. In summary, non-synonymous nucleotide substitutions unique to the phocids were generally concentrated in the loops between the A and B and the C and D helices.

Across terrestrial phyla the AB and CD loops are reported to have lower conservation than the conserved helices [24]. Indeed, crystal structure analysis of human leptin has shown that the AB loop forms a low density region that permits conformational change on binding with the receptor. In terrestrial mammals the CD loop is specifically conserved at residues Leu 104, Leu 107, Leu 110, Leu 114 and Val 113, which help to form a hydrophobic cap to protect the lipophilic residues at the surface of the BD helices [24]. With the exception of the Leu 104 these same residues in the CD loop are conserved within the phocids, also suggesting the retention of similar structure and function of the CD loop in these marine mammals.

Despite the sequence divergence of seal leptin, the ability to bind to LEPR has likely been conserved. Mutagenesis studies have revealed the critical residues for receptor binding and signalling in leptin form a hydrophobic cleft (Figs. 1 and 3B) [25], [26]. These positions, within the A and C helices, have been conserved within the seal and all mammalian leptins. The single amino acid differences at positions 15 and 85 in each seal species are conservative and do not affect the hydrophobic cleft formation necessary for binding [25], [26] (Fig. 3B). Residues 4, 5, 8, 11, 13, 78, 79, 81 and 89, which surround the hydrophobic cleft, are all predicted to be buried within the leptin and LEPR interface and are highly conserved in all mammals including the phocids. The one exception is the alanine at residue 81 which is unique to the phocids: all other mammals have a glutamic acid at this position. This residue is on the periphery of the binding site and considering the overall amino acid conservation it is likely that the seal leptin binds LEPR in the same manner as other mammals, including the California sea lion. However, in comparison to other marine and terrestrial mammals, residue substitution within phocid leptin does alter the predicted surface electrostatic potential – especially at locations away from the hydrophobic cleft (Fig. 3C). These substitutions considerably influence the relative isoelectric points of the different mammalian leptins. Whilst these predictions emphasize the evolved differences between phocid and other mammalian leptins since divergence, the significance of this to the interaction between leptin and the LEPR awaits experimental verification.

**Figure 4:**
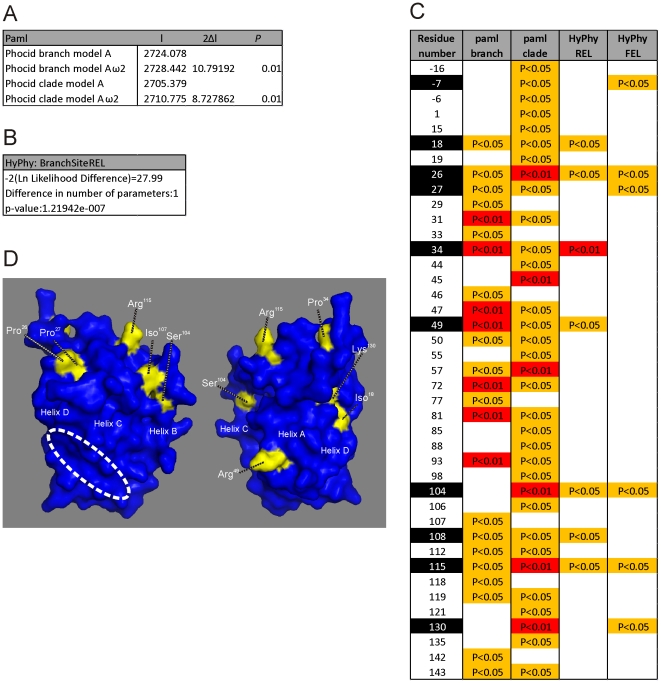
Positively selected residues in the phocid seal leptin occur away from the LEPR binding cleft. Likelihood ratio test statistics generated from the PAML branch model (A) and the REL analysis implemented using HyPhy (B) using the phocid branch of the tree. C shows the residues predicted to be under positive selection and statistical support by Bayes Empirical Bayes implemented in PAML and HyPhy REL and FEL analysis. D. Two views of the grey seal leptin with the selected residues identified in yellow and individually labelled. The positions of the A, C and D helices are indicated. The position of the hydrophobic binding cleft is identified with the white ellipse.

The large number of non-synonymous substitutions in the seal leptin cDNA compared to other mammalian species suggests that natural selection rather than genetic drift has influenced these genes. The three phocid cDNA sequences have a dN/dS ratio (the average rate of nonsynonymous substitutions/the average rate of synonymous substitutions) of 2.8. A value greater than >1 suggests positive selection. The branch model implemented in PAML [27] and both the REL and FEL tests implemented in HyPhy [28] consider condon variation in dN/dS and found strong evidence of positive selection for phocid leptin cDNA sequences alone (p<0.01) and along the branch leading to the phocid clade (p<0.01) (Fig. 4). In addition, several individual residues were considered to have evolved under positive selection with high confidence (p<0.05) throughout the molecule. We are only considering codons that reached a significance level of p<0.05 using two independent tests of selection as under positive selection. These are positions −7, 18, 26, 27, 34, 49, 104, 107, 115 and 130 (Figs. 1 and 3C). All of these selected residues lie away from the hydrophobic binding cleft, or in the case of −7, are in the signal peptide. It should be noted that with a larger sequence dataset it is likely that more residues would be considered to have evolved under positive selection that were approaching significance in this stringent analysis. Analysis of the cetacean sequence clade and the branch leading to this cluster only identified a single residue, Thr^46^, occurring within the variable AB loop, as being under weak positive selection, as has been described previously [20].

### The leptin binding site of the grey seal leptin receptor (LEPR) is conserved

**Figure 5:**
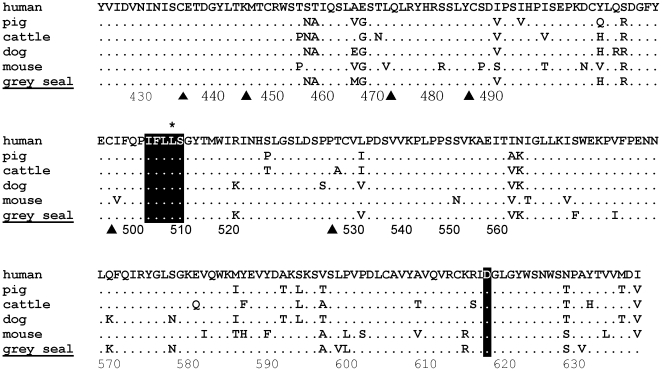
Conservation of the CRH2 region of the grey seal leptin receptor (LEPR) with selected terrestrial mammals. Identical residues to human LEPR are marked with a dot and residue numbering is according to human LEPR. Shading represents the residues critical for leptin binding, cysteine residues critical for folding are indicated by ▴and the most crucial residue for leptin binding is indicated by a ‘*’ above the alignment [24], [25]. The grey seal LEPR sequence reported in this study is underlined.

The main leptin affinity site of human LEPR is the cytokine receptor homology module 2 (CRH2) and mutagenesis has revealed the residues critical for leptin binding [26], [29]. The CRH2 domain of the seal LEPR was cloned as part of a larger cDNA fragment from the lung of a grey seal. Alignment of the predicted amino acid sequence with other mammal sequences representing diverse phyla reveals similar sequence conservation between all species (Fig. 5). All of the residues essential for leptin binding are identical between species and the seal leptin binding interface is intact. Evidence for sequence conservation of for the CRH2 region of the LEPR provides additional support for the conclusion that the leptin LEPR binding within the phocid seals is functionally retained.

## Discussion

We found that a large number of non-synonymous substitutions in phocid seal leptin have been driven by positive selection and are unique to this Family of marine mammals. This is in broad agreement with previous studies, although our stringent comparative analysis identified fewer residues under selection [20]. We detected very limited evidence for positive selection within the wider pinniped or the Carnivora clades; thus the diversification seen in phocid seal leptin has occurred since their divergence from the otariids. In comparison, we found very limited evidence of positive selection in cetaceans in contrast to other recent studies [20]. This disparity is likely caused by the different sequences analysed and the criteria used to determine selection. However, our results show that in comparison to cetacean leptin, the phocid leptin gene has been under stronger and more focussed selection over a considerably shorter evolutionary timespan.

These analyses indicate that the positive selection of phocid leptin occurred away from the LEPR binding region. Amino acid substitutions were generally concentrated in the variable AB loop and did not affect the formation of the four helices that are essential to the overall tertiary structure of a functional molecule. The predicted protein retains a hydrophobic cleft necessary to bind to the LEPR; the LEPR itself shows little evidence of residue substitution and no evidence of positive selection. However, whilst substitutions identified in this study do little to affect tertiary structure or hydrophobicity of the predicted LEPR binding cleft, the electrostatic potential of the binding surface is more neutral than that predicted for human and other marine mammal leptins. Outside of the hydrophobic cleft the surface electrostatic potential, and overall molecular isoelectric point (pI), of the phocid leptins is markedly different to other marine and terrestrial mammals (Fig. 3C). It would appear that the main consequence of seal leptin sequence divergence, at least partially driven by positive selection, has been to alter the electrostatic potential and pI. It is unclear precisely what the functional consequences of this would be, but differences in the net charge of mammalian orthologs can be associated with changes in protein interactions and function. Moreover, significant pI changes between orthologous proteins of human and mouse are concentrated in genes associated with hormone activity and cytokine activity, both directly applicable to leptin [30]. Overall we conclude from these analyses that the interaction between phocid leptin and LEPR remains intact. Because the receptor binding site is conserved and the receptor itself is also conserved, we conclude that phocid leptin is a functional hormone although the kinetics of leptin LEPR binding are likely to affected by the change in surface electrostatic potential of leptin in the phocids. It is probable that the evolution of seal leptin has, to some extent, altered receptor interaction. Unquestionably however, these conclusions on interactions and kinetics would have to be confirmed experimentally in seal-specific binding assays.

The phylogenetic placement of phocid leptin correlates with the major functions it fulfils in respiratory and metabolic physiology which are both very different in phocid seals compared to many other marine mammals. It has been hypothesized that the selection pressure on leptin in the pikas and bats was driven by cold environmental stress [18], [19]. However, although many phocid species are inhabitants of extremely cold environments, such as Arctic and Antarctic waters, their subcutaneous blubber layer means they are exceptionally well insulated against the cold. All marine mammals are homeotherms that maintain normal mammalian blood temperatures and the maintenance of a subcutaneous layer of insulative fat is clearly an important thermal adaptation. These species do not produce additional heat through thermogenesis in the same way as small mammals so it seems unlikely that this is the reason for the evolution in phocid leptin.

Phocid seals are well adapted to fasting for prolonged periods and during lactation can meet their own energy requirements as well as feeding a pup entirely from their stored fat reserves. Since leptin plays a critical role in the long term management and integration of energy reserves it seems that this may be a mechanism by which seal leptin could have evolved. However, many phocids do not exclusively fast during lactation. For example Weddell seals often feed after parturition [31], but have a divergent leptin gene and dive to great depths. Harbour seals also feed during late lactation [32] as do two other phocid seal species; harp seals (*Phoca groenlandica*) [33] and ringed seals (*Pusa hispida*) [34]. In this respect they are therefore less dissimilar to otariids than was first thought. Although not conclusive, these physiological comparisons do seem to indicate that leptin's role in surfactant production may have been the dominant selective pressure diversifying phocid leptin. The function of leptin in the lung of the foetus is to provide additional surfactant when the lungs need to fill with air for the first time [7]. However, adult phocid seals need to switch additional surfactant production on and off when they spend prolonged periods on land, such as during moulting and breeding. Whilst the surfactant pathway without leptin signalling is sufficient for surfactant production for lung compliance and efficient gas exchange whilst on land, additional surfactant may be required for effective lung inflation following dives and lung collapse under pressure [35]. These signalling pathways will therefore not be developmental but will be physiological signals. The key difference between foetal lung inflation and phocid seal lung inflation is that the regular switch “off” is clearly important. The discrete regulation of leptin may therefore be completely different in these diverse marine mammal species with contrasting lung structures and behaviours.

In conclusion, whilst further experimental evidence is needed to confirm our functional hypothesis that phocid seal leptin has undergone positive selection to meet the selection pressure of repeated lung collapse by diving on expiration, it appears the phocids have evolved unique respiratory modifications at the molecular and morphological level that enable them to fully exploit the extremes of the marine environment.

## Methods

### Isolation of pinniped and cetacean leptin and LEPR cDNA sequences

Total RNA was extracted from blubber and lung tissue samples obtained from freshly dead marine mammals as detailed in Hammond et al. [16]. Blubber was collected from a freshly dead harbour porpoise (*Phocoena phocoena*) by-caught in a fishing net in St Andrews Bay, Scotland. California sea lion (*Zalophus californianus*) blubber was donated by the Marine Mammal Center, Sausalito, California from a stranded animal immediately following euthanasia, and Weddell seal blubber was obtained from a freshly dead pup during fieldwork at McMurdo Sound in conjunction with Antarctia New Zealand and Macquarie University, Australia. Lung tissue for LEPR sequencing was obtained from a freshly dead adult grey seal that stranded in St Andrews Bay, Scotland. All samples were stored in RNALater (Ambion, Cambridgeshire, UK) and kept at −20°C until RNA extraction was performed. Samples were homogenized with 3 ml of Tri-Reagent (Sigma, Poole, UK) before extraction according to the manufacturer's guidelines. After washing, the RNA was dissolved in DEPC-treated water and quantified using a spectrophotometer at 260 nm absorption.

Reverse transcription was performed with Superscript II M-MLV (LifeTechnologies, Paisley, UK) using 5 µg of denatured RNA (70°C for 10 min) from each of the samples according to the manufacturer's guidelines with the inclusion of 40 U of RNase inhibitor (Sigma, Poole, UK) in a 20 µl reaction. This reaction was then diluted 1∶3 with tricine-EDTA buffer before use as a template in PCR. To amplify both leptin and LEPR, degenerate primers were synthesized corresponding to conserved regions of their mRNA sequences from other mammalian species publically available. Leptin primer sequences successful for each species were as follows California sea lion: sense 5′CCCRASAAGCACAKCCKGG3′ and antisense 5′GGGYCMGGATAAAGGACAC3′, for the Weddell Seal; sense 5′TRTGTTGAAGCTGTGCCAATC3′ and anti-sense 5′TGAGGATCTGTTGGTAGATGGC3′ and for the harbour porpoise; sense 5′AGTCCAGGATGACACCAAAACC3′and antisense 5′GYTCAGRGCCACCACCTCYGT 3′. For amplification of the grey seal LEPR the sense primer was 5′CTTYCTYTTGCCTGCTGGA3′ and the antisense was 5′GTTAAGTASCCRTCAGTTTCAC3′. Standard PCR was performed using each primer pair at a concentration of 2 µM using Taq (Amersham, Buckinghamshire, UK) according to the manufacturer's guidelines in 20 µl reactions. PCR using primers for β-actin was also performed on each of the templates to confirm the integrity of the cDNA. Samples were run on 2% agarose gel containing ethidium bromide and amplified fragments were extracted (gel extraction kit; Qiagen, West Sussex, UK) and cloned using the pCR 2.1 TOPO vector (Life Technologies). Colonies were screened using M13 primers for an insert of the correct size. The plasmids of positive colonies were extracted (FastPlasmid™; Eppendorf, Cambridge, UK) and sequenced (Dundee Sequencing Service, University of Dundee). For rapid amplification of the leptin cDNA ends a set of primers for each sequence obtained from degenerate PCR was synthesized for 5′ and 3′ RACE. To amplify the region of LEPR predicted to bind leptin, primary and nested primers were synthesized for use only in 3′ RACE; LEPRGSP2-5′CCGAGCCAGTATACCGTGGTGGGTG3′ and the nested primer LEPRNGSP2-5′CAGGAATGCCACCATCGCTATGC 3′. Templates for each reaction were synthesized using Superscript II (Invitrogen, Paisley, UK) and the SMART RACE kit (BD Biosciences, Oxford, UK) in 10 µl reactions including RNase inhibitor. PCR cycling was performed at 69°C with the SMART RACE universal primers (UPM) supplied according to the manufacturers guidelines for 35 cycles using advantage cDNA polymerase (BD Biosciences, Oxford, UK) in 25 µl reaction volumes. For the LEPR nested reaction, 5 µl of the primary reaction was removed; diluted 1∶10 in water and 1 µl was used in an identical reaction substituting the UPM with the nested RACE primer. Samples were visualized on a 1.5% agarose gels, cloned and sequenced as above.

### Phylogenetic analysis and tests of selection

The marine mammal leptin sequences determined in this work were compared with the previously published complete cDNA sequences for the harbour (AJ618981) and grey (AJ618982) seal as well as a selection of mammalian leptin cDNA sequences available on GenBank (Figure S1). Sequences from this study are deposited in GenBank with the accession numbers AM157372 (California sea lion leptin), AM157373 (Weddell seal leptin), AM157370 (harbour porpoise leptin) and HM448474 (grey seal leptin receptor).

Sequences were aligned using CLUSTAL X and manually as necessary using Bioedit version 7.0.5.3 [36]. Neighbour-joining and maximum likelihood with nearest-neighbour-interchange phylogenetic analyses were performed with MEGA version 4 [36] using the cDNA sequences and pairwise deletion. The Tamura-Nei, Tamura 3-parameter, Tajima-Nei (Neighbour-Joining method only) and Kimura 2-parameter models were implemented for each method with 1000 replicates.

Estimation of the average rate of nonsynonymous substitutions/the average rate of synonymous substitutions (dN/dS) (ω) ratios was performed by maximum likelihood implemented in PAML, v3.14 [27]. We tested for evidence of variation in evolutionary rate alone different phylogenetic tree branches using the branch model within PAML. Likelihood ratio tests were performed to compare the likelihood of a tree topology given a null model that does not allow ω>1 for the branch of interest to the likelihood of the same tree topology given an alternative model that does using the F3×4 model of codon frequencies. The log-likelihood of the branch model was compared and a χ^2^ test was applied to twice the difference between the log ratios. A Bayes empirical bayes was used to identify codons with ω>1 [37]. To support and these analyses we implemented another Random Effects Likelihood method (BranchSiteREL.bf) available within the HyPhy package [37] that indicates lineages within a tree that have been under episodic selection. We also used a Fixed Effects Likelihood approach using the entire dataset to determine global alignment parameters implemented through QuickSelectionDetection with the HyPhy package using default model, estimated dN/dS parameters using one rate Fixed Effects Likelihood and full site-by-site Likelihood Ratio Test [28]. All tests were performed in triplicate and the pika branch and clade were used as a positive control for the methodology.

### Tertiary structure modelling

Homology modelling of grey seal and California sea lion leptin cDNA sequences were performed using the web based LOOPP and Swiss-Model suites [38], [39]. Hydrophobicity was calculated using the color_protscale module of rTools (Kristian Rother: www.rubor.de) and the Adaptive Poisson-Boltzmann Solver (APBS) – Software for evaluating the electrostatic properties of nanoscale biomolecular systems [40] and implemented and visualised using PyMol Molecular Graphics System, version (Schrödinger, LLC, Portland, OR).

## Supporting Information

Figure S1 **CLUSTAL X alignment of diverse mammalian leptin nucleotide sequences which was manually edited using Bioedit version 7.0.5.3** [**36**] **.** Alignment used as a basis from which to compare phylogenetic topologies using MP, ML and NJ methods. A representative tree generated using NJ prediction is shown in manuscript Fig. 2.
(TIF)
